# INTERGENERATIONAL RELATIONS OF MARRIED PERSONS WITH THEIR PATRILINEAL AND MATRILINEAL PARENTS IN CHINA

**DOI:** 10.1093/geroni/igad104.1743

**Published:** 2023-12-21

## Abstract

**Objective:**

Guided by modernization theory and intergenerational solidarity theory, this study examined the general patterns of intergenerational relations with patrilineal and matrilineal parents in contemporary China and explored its influencing factors and mechanism.

**Methods:**

Data from the 2017 Chinese General Social Survey were examined. Analytic samples consisted of married respondents with at least one patrilineal parent and one matrilineal parent alive (N = 1098). Latent class analysis (14 functional, structural and associational indicators) classified the intergenerational relations and multinomial logistic regression explored how these intergenerational relation patterns were associated with education, and marital and kinship relation by gender.

**Results:**

Five intergenerational relation types were revealed, three types (close to both parents and support upward more, 35.7%; close to both parents and reciprocal, 26.4%; distant to both parents, 11%) showed balanced patterns and two types (close to patrilineal parent but distant to matrilineal parent, 20%; close to matrilineal parent but distant to patrilineal parent, 6.9%) with unbalanced patterns. Children with higher educational level tended to form more egalitarian and bilateral intergenerational relations, both for husbands and wives. Moreover, good marital relation and more brothers facilitated this tendency for wives only.

**Conclusions:**

Results provided evidence for the maintenance and changes of patrilineal culture, and the increased importance of marital relation in China. Findings reaffirmed that married children had different interaction patterns with their patrilineal and matrilineal parents, and discussed the changes in social norms governing the responsibilities of husbands and wives. Keywords: intergenerational relations, patrilineal/matrilineal parents, latent class analysis, gender, China

